# A DTMOS-Based Memristor Emulator Circuit for Low-Power Biomedical Signal Conditioning

**DOI:** 10.3390/mi17030328

**Published:** 2026-03-05

**Authors:** Imen Barraj

**Affiliations:** Department of Computer Engineering, College of Computer Engineering and Sciences, Prince Sattam Bin Abdulaziz University, Al-Kharj 11942, Saudi Arabia; i.barraj@psau.edu.sa

**Keywords:** memristor emulator, DTMOS, pinched hysteresis loop, biomedical front-end

## Abstract

This paper presents a novel, minimalist floating memristor emulator circuit designed for low-power biomedical analog front ends. The proposed topology requires only two dynamic threshold MOS (DTMOS) transistors and one capacitor, constituting one of the most compact memristor emulators reported. The circuit operates without static power consumption and exploits the body-effect coupling in DTMOS devices to generate a state-dependent resistance. Comprehensive simulation in a 0.18 μm CMOS process verifies core memristive characteristics: a frequency-dependent pinched hysteresis loop tunable via capacitance, non-volatile memory, and robustness across temperature and process variations. Experimental validation using a discrete CD4007-based prototype confirms the pinched hysteresis loop from 100 Hz to 800 kHz, with a maximum simulated operating frequency of 500 MHz. A comparative analysis demonstrates that the design achieves a favorable trade-off, simultaneously minimizing transistor count and power while providing floating operation and high-speed performance. These attributes make the emulator a compelling candidate for integration into adaptive, area and power constrained biomedical signal conditioning systems.

## 1. Introduction

Wearable healthcare systems require analog front-end circuits that are power-efficient, compact, and capable of advanced signal conditioning. These circuits must reliably acquire weak, noisy biosignals such as electrocardiogram (ECG), electroencephalogram (EEG), or electromyogram (EMG), under strict physical and energy constraints. Conventional linear circuits, built from operational amplifiers and passive components, often lack the adaptive behavior needed for dynamic noise filtering or intelligent gain control without increasing complexity and power drawing. This challenge has motivated the exploration of neuromorphic engineering, which mimics the efficient, adaptive nature of biological neural processing. A key element in this field is the memristor, a theoretically predicted passive component whose resistance depends on the history of the time integrals of current (charge) and voltage (flux), making it inherently adaptive and memory driven [1].

Despite their promise, physical memristors are not yet widely available in standard semiconductor processes [2]. As a practical alternative, memristor emulators built from conventional CMOS components allow researchers to explore novel circuit architectures today. Most existing emulator designs, however, rely on multiple active blocks such as operational transconductance amplifiers (OTAs) or analog multipliers [3,4,5,6]. These designs tend to consume significant power, occupy large areas, and require careful tuning, making them suited for ultra-low-power biomedical applications. There remains a clear need for a simpler, more integrable memristor emulation core that aligns with the demands of wearable and implantable medical electronics.

Recent research in memristor emulation has strategically pivoted toward minimalist, transistor-efficient designs that reduce complex active components. The objective is to create emulator circuits that are inherently suitable for low-power, low-voltage operation and easier integration into system-on-chip designs, particularly for portable applications. This movement is characterized by a deliberate effort to minimize the number of active devices, leveraging instead the intrinsic nonlinearities of transistors operating in specific regimes. Several notable designs exploit MOSFETs in the subthreshold region to achieve the necessary exponential current–voltage relationships with minimal static power. Other approaches utilize the body terminal as a control node, as seen in designs employing the dynamic threshold MOS, DTMOS transistor. Babacan et al. introduced a grounded emulator topology requiring only four transistors, though its electronic tunability is constrained [7]. Similarly, a compact floating emulator employing four transistors was reported, yet it lacks experimental verification and adjustable memory characteristics [8]. A significant advance in high-frequency performance was achieved by Zhou et al., whose two-transistor floating emulator operates effectively up to 300 MHz [9]. Additionally, alternative pathways utilizing passive and diode-based topologies have been explored. Corinto and Ascoli, for example, realized a floating memristive cell by integrating an RLC network with four diodes [10]. A recurring constraint across several of these minimalist designs [7,10,11,12] is the absence of a simple, integrated method for the electronic adjustment of memductance. Other implementations prioritize functional accuracy over component count, as seen in Saxena’s design, which utilizes seven transistors and a biasing source to produce a well-defined pinched hysteresis loop at the cost of increased circuit complexity [13].

Consequently, a highly attractive design methodology has emerged: constructing emulators exclusively from MOSFETs and a minimal complement of passive elements. This approach offers the compelling advantages of enhanced operating bandwidth, simplified layout, and reduced static power dissipation. Notable examples following this philosophy include the floating emulator by Vista and Ranjan, which combines three NMOS transistors with a capacitor and a DC source for operation up to 13 MHz [14], and the fully passive, non-ideal memristor proposed by John et al., implemented with BJTs, diodes, capacitors, and resistors for kilohertz-range operation [15]. Concurrently, the theoretical modeling of memristive devices is evolving, with contributions such as those by E. Gale extending the framework for non-idealities, including detailed descriptions of physical mechanisms like filamentary switching [16,17]. The refinement of CMOS-specific emulator circuits remains an active focus. Koymenn and Emmanuel developed a memristor exploiting weak-inversion operation, utilizing two log-domain transconductors and a grounded capacitor [18]. Efforts toward greater compactness are illustrated in Vishal’s design, which employs seven MOS transistors, with specific types including PMOS, NMOS, and ZVT NMOS, alongside an external DC source [19]. While the efforts in recent literature [20] strongly favors reducing transistor count, these ultra-minimalist circuits frequently encounter inherent trade-offs involving silicon area, power efficiency, maximum frequency, and fabrication process sensitivity.

This paper introduces a novel floating memristor emulator circuit designed to directly address this need for integration low-power emulation. The proposed circuit, named the DTMOS-based memristor emulator (DMEC), is built around a minimalist topology requiring only two dynamic threshold MOS transistors and a single capacitor. In this configuration, the DTMOS devices, where the gate and body terminals are connected, leverage their inherent voltage-dependent threshold to generate the essential nonlinear resistance modulation. The capacitor serves as the integrating element, storing the state variable and ensuing the hysteretic feedback loop that defines memristive memory. By eliminating multi-transistor active blocks like OTAs or multipliers, this architecture achieves a significant reduction in both component count and static power overhead, making it inherently suitable for low-voltage operation and dense integration in system-on-chip designs.

The remainder of this paper is organized as follows. Section 2 details the proposed emulator circuit, presenting its topology, operating principle, and comprehensive mathematical analysis. Section 3 discusses the simulation and experimental results, validating the memristive characteristics and robustness of the design. Section 4 presents a comparative analysis, discussion with prior works, and suggests directions for future research. Finally, Section 5 concludes the paper and summarizes the key contributions.

## 2. The Proposed Memristor Emulator Circuit

This section details the proposed DMEC, outlining its topology and providing a foundational mathematical analysis to describe its operating principle.

**Figure 1:**
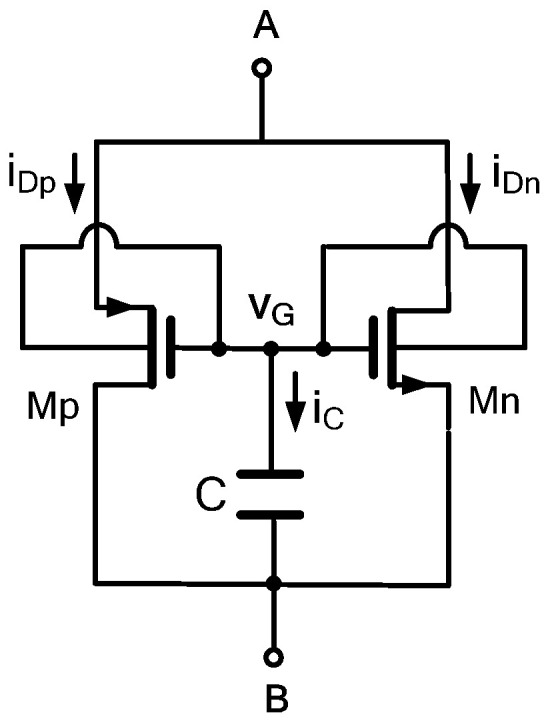
Proposed DTMOS-based memristor emulator.

The schematic of the proposed DMEC is illustrated in Figure 1. The core of the circuit is a minimalistic and symmetrical structure employing two DTMOS transistors (Mp and Mn) and a single capacitor (C). The two DTMOS devices are configured in a complementary push–pull arrangement: Mp is a P-type (PMOS) device, and Mn is an N-type (NMOS) device. The interconnection forms a floating two-port network with terminals labeled A and B, which serve as the input/output ports for the memristive element. The topology is defined by the following critical connections: the source terminal of the PMOS-Mp and the drain terminal of the NMOS-Mn are connected to terminal A; the drain terminal of the Mp and the source terminal of the Mn are connected to terminal B; the gate terminals of both transistors are tied together, forming a common control node; and the capacitor C is connected between this common gate node and terminal B.

In this configuration, the capacitor serves as the state variable element, with its voltage ($V_{G}$) representing the internal memory of the memristor. The complementary DTMOS pair (Mp, Mn) acts as a voltage-controlled nonlinear resistor. They perform two essential functions: (1) they convert the applied voltage ($V_{AB}=V_{A}-V_{B}$) into a current that charges or discharges capacitor C and (2) they modulate the effective resistance between terminals A and B based on the stored state $V_{G}$. The dynamic threshold behavior of the transistors, where the body is tied to the gate, is key to generating the necessary nonlinear, state-dependent current–voltage relationship. The fundamental operation of the DMEC can be derived from the DTMOS transistor characteristics. For a DTMOS device, the gate-to-body connection modifies the threshold voltage ($V_{TH}$) to become a function of the gate-source voltage. The threshold voltage for the DTMOS devices, which governs their conduction, is given by [21]:

(1) $$V_{TH}=V_{TH0}+\gamma \left(\sqrt{\left|2\phi_{F}-V_{SB}\right|}-\sqrt{\left|2\phi_{F}\right|}\right)$$

where $V_{TH0}$ is the zero-bias threshold voltage, γ is the body-effect coefficient, $\phi_{F}$ is the surface potential, and $V_{SB}$ is the source-to-body voltage. For the DTMOS configuration, $V_{SB}$ is intrinsically linked to the gate potential, creating the desired feedback mechanism between the state variable $V_{G}$ (across capacitor C) and the channel conductivity.

The operating principle of the proposed DMEC can be explained by analyzing the conduction cycles of the complementary DTMOS pair, Mp and Mn. For analysis, consider a sinusoidal voltage $v_{AB}(t)=v_{A}-v_{B}$ applied across the floating terminals. Let $v_{C}$ denote the voltage across the state capacitor C, which is equivalent to the common gate-to-terminal B voltage ($V_{G}$).

**Figure 2:**
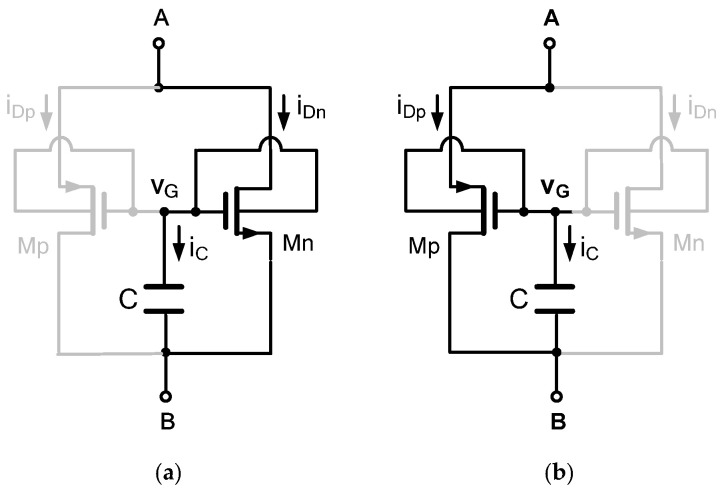
DMEC states of (**a**) positive and (**b**) negative half-cycle.

The generation of the pinched hysteresis loop in the proposed emulator stems from the complementary and asymmetric body-effect modulation of the two DTMOS transistors. During operation, Mp and Mn conduct alternately over the input cycle. In the positive half-cycle, terminal A is at a higher potential than B. In this phase, the Mp transistor is in a cut-off or high resistance state, while the Mn transistor is active, as shown in Figure 2a. The capacitor voltage $v_{C}$ dynamically adjusts the threshold voltage $V_{THn}(v_{C})$ of Mn through the DTMOS body effect. As $v_{A}$ increases, $v_{C}$ also rises but with a phase lag, effectively reducing the overdrive voltage $\left(v_{C}-V_{THn}\right)$ as the cycle progresses. This results in a decreasing channel resistance with increasing input voltage, producing a negative, counterclockwise, lobe in the $i_{AB}$-$v_{AB}$ characteristic. Conversely, in the negative half-cycle, terminal B is at a higher potential than A. The roles of the transistors reverse. The Mn is now off, and the Mp becomes active, as depicted in Figure 2b. Here, the body-effect of the Mp transistor operates in the opposite manner; the modulating $v_{C}$ now causes the Mp channel resistance to increase with the magnitude of the negative input voltage, yielding a positive lobe. This alternating action creates the complete pinched hysteresis loop, where the pinch-point at the origin is preserved as both transistor currents approach zero when $v_{AB}=0$. The observed lobe polarity is a direct consequence of the DTMOS architecture, where the state capacitor voltage couples into the transistor threshold in opposite ways for NMOS and PMOS devices. Thus, under a periodic input signal, Mp and Mn conduct alternately, controlling the charge and discharge of capacitor C and causing $v_{C}$ to vary periodically with time. Crucially, in the DTMOS configuration, the capacitor voltage $v_{C}$ directly controls both the gate-source voltage and the body-source voltage of each transistor. This dual control via the dynamic threshold effect creates a strong, nonlinear dependence of the transistor’s channel resistance on the state variable $v_{C}$. The equivalent memristance between terminals A and B is the series combination of the dynamically modulated resistances of Mp and Mn as they alternate conduction, resulting in the characteristic pinched hysteresis loop.

The proposed DMEC operates with complementary conduction: Mn is active during positive half-cycles (V_AB_ > 0), while Mp is active during negative half-cycles (V_AB_ < 0). Our analysis reveals that the active transistor in each half-cycle traverses three distinct operating regions. Linear region operation dominates the mid-range of each half-cycle, where the voltage–current relationship is most linear and the memristance modulation is strongest. As the input voltage approaches its peak, the transistor enters the saturation region, where V_DS_ exceeds the overdrive voltage. Near the zero-crossing, the transistor operates in subthreshold conduction, with V_GS_ close to V_TH_ and currents exponentially small. During the opposite half-cycle, the transistor is in cut-off, ensuring proper complementary operation of the push–pull configuration. The mathematical derivation presented in this section employs the linear region approximation as a simplifying assumption to obtain analytical insight into the memristive behavior. This approximation is justified for several reasons. First, the linear region captures the essential voltage-controlled resistance behavior that underlies the memristive effect. In the linear region, the drain current is proportional to V_DS_, creating the direct relationship between voltage and current that enables memristance modulation. Second, the contribution from the saturation regions occurs primarily at the lobe tips of the hysteresis loop. In saturation, the current becomes relatively independent of V_DS_, which affects the exact shape of the lobe tips but does not alter the fundamental memristive characteristics, pinched hysteresis, frequency dependence, and state retention. Third, subthreshold conduction near the zero-crossing has negligible impact on the overall behavior, as currents in this region are exponentially small. Consequently, despite the presence of saturation and subthreshold regions, the core memristive behavior is preserved. The memristor’s defining fingerprint, pinching at the origin, is maintained because the current approaches zero as the voltage approaches zero.

Because the circuit operation is symmetrical between the positive and negative half-cycles, the analysis for one half-cycle suffices. Consider the positive half-cycle with terminal B as the reference, ground. Additionally, the two DTMOS transistors Mp and Mn operate alternately in the linear region to modulate the channel resistance, controlled by the state voltage $v_{C}$ across capacitor C. For analysis, as the terminal B is taken as ground reference ($v_{B}=0$), thus $v_{AB}=v_{A}$ and $v_{C}=v_{G}$. The threshold voltage for the Mn transistor is as follows:

(2) $$V_{THn}(v_{C})=V_{TH0,n}+\gamma_{n}(\sqrt{2\phi_{F,n}+v_{C}}-\sqrt{2\phi_{F,n}})$$

For small $v_{C}$, this can be linearized using a first-order Taylor approximation, as follows:

(3) $$V_{THn}\left(v_{C}\right)\approx V_{TH0,n}+\alpha_{n}v_{C},\;\;\mathrm{where}\;\alpha_{n}=\frac{\gamma_{n}}{2\sqrt{2\phi_{F,n}}}$$

A similar expression holds for the PMOS (M1) with parameters $V_{TH0,p}$ and $\alpha_{p}$.

The transistors are assumed to operate in the linear region when active. Thus, the drain current for Mn can be expressed as follows:

(4) $$i_{Dn}=k_{n}\left[(V_{GSn}-V_{THn})V_{DSn}-\frac{V_{DSn}^{2}}{2}\right]$$

where $k_{n}=\mu_{n}C_{ox}(W/L{)}_{n}$, $V_{GSn}=v_{C}$, and $V_{DSn}=v_{A}$ during the positive half-cycle.

The core of the proposed DMEC behavior is the dynamics of the state voltage $v_{C}$. The capacitor C is charged or discharged by a current that is a function of the terminal voltage $v_{A}$ and the state itself. This current originates from the body-effect coupling in the DTMOS structure.

During the positive half-cycle ($v_{A}>0$), Mn is active. The change in $v_{C}$ is governed by the capacitive current flowing into the common gate/body node. This current is proportional to the time derivative of the threshold voltage, which depends on $v_{C}$ through the body effect. Applying charge conservation at the gate node yields the following:

(5) $$C\frac{dv_{C}}{dt}=\beta_{n}\cdot k_{n}\left[(v_{C}-V_{THn}(v_{C}))v_{A}-\frac{v_{A}^{2}}{2}\right]$$

Here, $\beta_{n}$ (where $0<\beta_{n}<1$) is a coupling coefficient that accounts for the fraction of the channel current that contributes to changing the body/gate potential via the body-source capacitance and the forward-biased body-source diode in the DTMOS configuration. This term links the terminal current to the state evolution, a key feature of memristive systems. Substituting the linearized threshold voltage Equation (3) into Equation (5) gives the following:

(6) $$C\frac{dv_{C}}{dt}=\beta_{n}k_{n}[((1-\alpha_{n})v_{C}-V_{TH0,n})v_{A}-\frac{v_{A}^{2}}{2}]$$

This is a first-order nonlinear differential equation for the state variable $v_{C}$ driven by the input $v_{A}$. The terminal current $i_{AB}$ during the positive half-cycle is the drain current of Mn, as follows:

(7) $$i_{AB}=i_{Dn}=k_{n}[((1-\alpha_{n})v_{C}-V_{TH0,n})v_{A}-\frac{v_{A}^{2}}{2}]$$

The memductance W is defined as $W=i_{AB}/v_{A}$. For a sinusoidal input $v_{A}(t)=Asin(\omega t)$, and assuming the amplitude A is small such that the quadratic term $v_{A}^{2}/2$ is negligible compared with the linear term, the memductance simplifies to the following:

(8) $$W\approx k_{n}\left[(1-\alpha_{n})v_{C}(t)-V_{TH0,n}\right]$$

This shows that the memductance is linearly controlled by the state voltage $v_{C}(t)$.

To find $v_{C}(t)$, we solve the state Equation (6). Under the same small-signal assumption and ignoring the $v_{A}^{2}$ term, Equation (6) becomes the following:

(9) $$C\frac{dv_{C}}{dt}\approx \beta_{n}k_{n}\left[(1-\alpha_{n})v_{C}-V_{TH0,n}\right]Asin(\omega t)$$

This is a linear time-varying differential equation. For high frequencies where $v_{C}$ cannot follow the instantaneous input, we look for a quasi-steady-state solution. Integrating over a half-cycle and considering the periodic nature, the average effect yields a solution where $v_{C}$ contains a DC component $V_{C0}$ and a phase-shifted component at frequency ω, as follows:

(10) $$v_{C}(t)\approx V_{C0}+\frac{\beta_{n}k_{n}AV_{C0}(1-\alpha_{n})}{C\omega }cos(\omega t)$$

where $V_{C0}\approx \frac{V_{TH0,n}}{\left(1-\alpha_{n}\right)}+\varepsilon$ is the equilibrium voltage around which $v_{C}$ oscillates and ε is a small offset determined by the circuit’s operating point.

Substituting Equation (10) into Equation (8) gives the following, final expression for memductance:

(11) $$W(t)\approx k_{n}\left[(1-\alpha_{n})V_{C0}-V_{TH0,n}\right]+\frac{k_{n}^{2}\beta_{n}AV_{C0}(1-\alpha_{n}{)}^{2}}{C\omega }cos(\omega t)$$

This can be written more compactly as follows:

(12) $$W(t)\approx W_{0}+\Delta Wcos(\omega t)$$

where $W_{0}=k_{n}\left[(1-\alpha_{n})V_{C0}-V_{TH0,n}\right]$ is the constant memductance offset and $\Delta W=\frac{k_{n}^{2}\beta_{n}AV_{C0}(1-\alpha_{n}{)}^{2}}{C\omega }\;$ is the amplitude of the time-varying memductance.

Equation (12) reveals the frequency-dependent behavior critical to memristor emulation. The time-varying component ΔWcos(ωt) is inversely proportional to the input frequency ω. Therefore, at low frequencies, ω is small and ΔW is large, resulting in a significant modulation of resistance within one cycle and a wide pinched hysteresis loop. As frequency increases, ΔW shrinks. The memductance variation diminishes. In the high-frequency limit (ω→∞), ΔW→0. The memductance converges to the constant $W_{0}$ and the emulator behaves as a linear resistor, $i_{AB}=W_{0}\cdot v_{A}$. This is consistent with the fundamental property that a memristor’s hysteresis lobe area decreases with increasing frequency.

Furthermore, to determine the time constant, we define the ratio of the dynamic part to the static part of the memductance. From Equation (11), the ratio of the time-varying part to the static part is as follows:

(13) $$\frac{\Delta W}{W_{0}}=\frac{k_{n}^{2}\beta_{n}AV_{C0}(1-\alpha_{n}{)}^{2}}{C\omega W_{0}}=\frac{1}{\tau f}$$

Substituting ω=2πf and using the approximation $W_{0}\approx k_{n}(1-\alpha_{n})V_{C0}$, where $V_{TH0,n}$ is neglected because $(1-\alpha_{n})V_{C0}\gg V_{TH0,n}$ under normal operating conditions, we obtain the following:

(14) $$\frac{1}{\tau f}=\frac{k_{n}^{2}\beta_{n}AV_{C0}(1-\alpha_{n}{)}^{2}}{C\cdot 2\pi f\cdot k_{n}(1-\alpha_{n})V_{C0}}=\frac{k_{n}\beta_{n}A(1-\alpha_{n})}{2\pi Cf}$$

Thus, the time constant can be expressed as follows:

(15) $$\tau =\frac{2\pi C}{k_{n}\beta_{n}A(1-\alpha_{n})}$$

During the negative half-cycle ($v_{A}<0$), the Mp becomes active. A parallel analysis using PMOS parameters ($k_{p},\alpha_{p},V_{TH0,p}$) yields a complementary expression for memductance. The alternating action of the NMOS and PMOS devices, each with its body-effect coefficient, generates the complete, symmetric pinched hysteresis loop characteristic of a floating memristor. The derived relationship $i_{AB}(t)=W(v_{C},t)\cdot v_{A}(t)$, where W is a function of the state variable $v_{C}$ which itself obeys a differential equation driven by $v_{A}$ and $i_{AB}$, satisfies the formal definition of a voltage-controlled memristor within the broader class of memristive systems. The analysis, therefore, provides a solid theoretical foundation for the emulator’s operation.

The mathematical derivation assumes linear region operation for the active transistor in each half-cycle. Detailed verification across the full 1.8 V peak input swing reveals that the active transistor operates in three distinct regions: linear region for approximately 45% of each half-cycle ($V_{AB}\;$ between 0.4 V and 1.2 V), saturation region near the voltage peaks ($V_{AB}$ > 1.2 V) for approximately 33% of the half-cycle, and subthreshold conduction near zero-crossing ($V_{AB}\;$ < 0.4 V) for approximately 22% of the half-cycle. Despite this complexity, the linear region approximation successfully captures the essential memristive behavior. The saturation regions primarily affect the exact shape of the hysteresis lobe tips but do not alter the fundamental memristive fingerprints, pinched hysteresis, frequency-dependent lobe contraction, and state-dependent resistance modulation. The subthreshold region near zero-crossing contributes negligibly to the overall behavior due to exponentially small currents.

## 3. Simulation and Experimental Verification

To validate the operational feasibility and performance characteristics of the proposed DMEC, this section presents comprehensive simulation and experimental analyses. The circuit was simulated using the advanced design system (ADS) tool with a standard 0.18 µm CMOS process to verify its memristive fingerprints, including the pinched hysteresis loop, frequency-dependent behavior, non-volatility, and tunability. The value of the capacitor is selected to match the targeted frequency range of operation, ensuring the circuit functions within its intended memristive regime. Furthermore, a physical prototype was implemented using discrete components to confirm the simulation results under practical conditions. These investigations collectively demonstrate that the proposed two-transistor, one-capacitor topology successfully emulates the defining characteristics of a floating memristor while offering advantages in simplicity, power consumption, and integration potential for biomedical front-end systems.

### 3.1. Numerical Analysis

**Figure 3:**
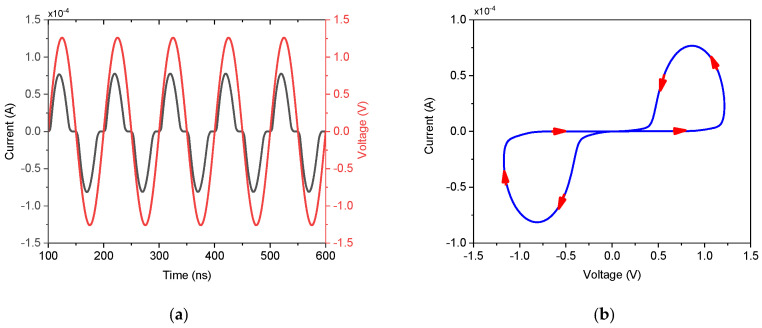
(**a**) Time-domain voltage and current response of the proposed DMEC at 10 MHz sinusoidal input and the (**b**) corresponding pinched hysteresis loop.

The signature fingerprint of a memristor, the pinched hysteresis loop in the current–voltage plane, was confirmed under sinusoidal excitation. Figure 3a shows the transient voltage $v_{AB}$ and current $i_{AB}$ waveforms for a 1.8 V peak, 10 MHz input signal, using a capacitor C = 50 pF and DTMOS transistors sized at ${\left(w/L\right)}_{Mp}=1\;\mu m/0.18\;\mu m$ and ${\left(w/L\right)}_{Mn}=0.5\;\mu m/0.18\;\mu m$. The current waveform exhibits a clear non-linearity and a measurable phase shift relative to the voltage, a direct consequence of the state-dependent resistance modulation by capacitor voltage $v_{C}$. Figure 3b plots the corresponding $i_{AB}$–$v_{AB}$ characteristic, displaying a well-defined, symmetric PHL that is pinched precisely at the origin ($i_{AB}=0$, $v_{AB}=0$). The lobe area is significant at this frequency, indicating strong memristive modulation. This result provides the first fundamental validation that the proposed two-transistor, one-capacitor topology successfully emulates the constitutive behavior of a floating memristor. Furthermore, as shown in Figure 3b, the hysteresis trajectory follows in a clockwise direction. For positive voltages, as voltage increases from 0 to +Vmax, the current follows the lower branch of the hysteresis loop. This indicates that the instantaneous memristance, the inverse slope of the I–V curve, is relatively lower during the increasing voltage phase, allowing current to increase at a faster rate. As voltage decreases from +Vmax back to 0, the current returns via the upper branch. This indicates that the memristance is relatively higher during the decreasing voltage phase, resulting in higher current at the same voltage levels compared with the increasing phase. For negative voltages, a symmetric clockwise pattern appears. As voltage becomes more negative, current follows the lower branch, more negative current, and as voltage returns to zero, current follows the upper branch, less negative current. Additionally, the clockwise direction provides insight into the internal dynamics of the proposed DMEC. During the increasing voltage phase, the capacitor voltage, $v_{C}$, builds up, modulating the threshold voltage of the DTMOS transistors via the body effect. This reduces the effective resistance, allowing more current to flow. During the decreasing voltage phase, the capacitor voltage remains elevated, due to its memory characteristic, maintaining the lower resistance state even as the input voltage decreases. This creates the hysteresis, the current is higher on the return path than it was on the forward path at the same voltage. At the zero-crossing, both branches converge precisely at the origin (0,0), confirming the memristor’s defining fingerprint. Therefore, for a given positive voltage, the current is higher when the voltage is decreasing than when it is increasing. This history-dependent behavior is the essence of memristive operation.

**Figure 4:**
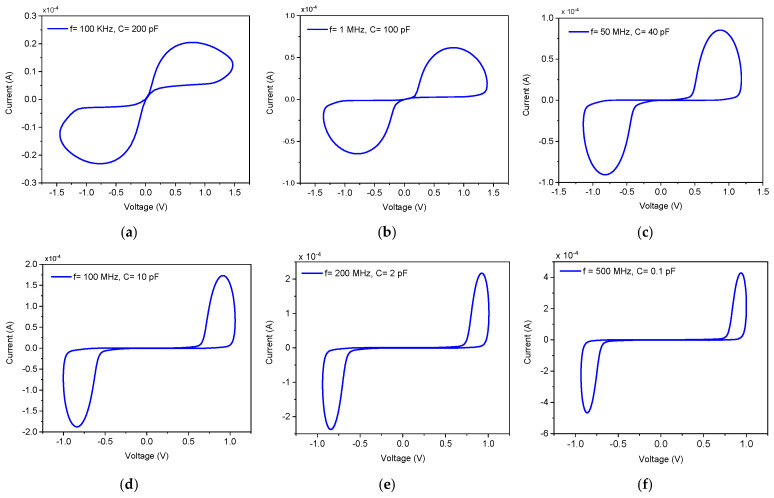
PHLs of the proposed DMEC for different frequencies of (**a**) 100 kHz, (**b**) 1 MHz, (**c**) 50 MHz, and (**d**) 100 MHz, (**e**) 200 MHz, and(**f**) 500 MHz.

A fundamental property of memristive systems is the contraction of the hysteresis lobe area with increasing input frequency, a direct consequence of the state variable’s finite response time. This was rigorously characterized by applying a fixed 1.8 V amplitude sinusoidal signal across a broad frequency spectrum from 100 kHz to 500 MHz, with the state capacitor C scaled from 200 pF to 100 fF in accordance with the derived time constant to maintain observable hysteresis. As shown in Figure 4, the PHL exhibits a systematic evolution: at 100 kHz with C=200 pF, the characteristic displays a pronounced hysteresis lobe, indicating strong memristive modulation. As frequency increases to 100 MHz, the lobe contracts. At 200 MHz, the current–voltage relationship approaches linearity, with the hysteresis lobe substantially diminished. By the maximum tested frequency of 500 MHz, the lobe area is further reduced to a minimal trace. As frequency increases further, the PHL converge to a single straight-line characteristic of a linear resistor, as the state capacitor becomes incapable of tracking the rapid input variations, thereby freezing the memristance at its time-averaged value. This frequency-dependent pinching aligns perfectly with the theoretical model expressed in Equation (11) and confirms the emulator’s dynamic conformity to ideal memristor behavior.

**Figure 5:**
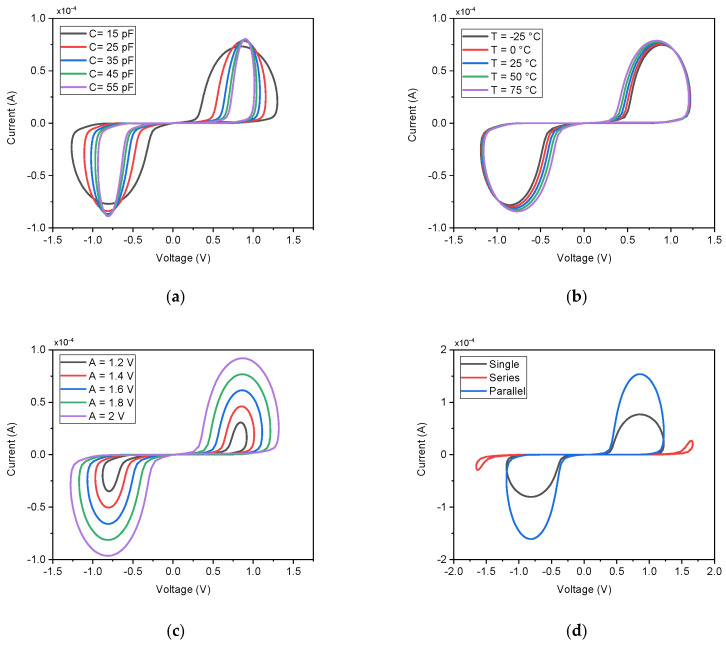
Performance characterization of the proposed DMEC: (**a**) PHL variation with capacitance, (**b**) temperature dependence, (**c**) input amplitude scaling, and (**d**) response in different circuit configurations.

A key advantage of the proposed DMEC topology is the straightforward electronic tunability of its memristance via the state capacitor C. This parameter provides direct control over the circuit’s time constant, thereby governing the rate of state change and the extent of hysteresis. Figure 5a illustrates the PHL for capacitance values ranging from 15 pF to 55 pF at a fixed excitation frequency of 10 MHz and amplitude of 1.8 V. As C increases, the hysteresis lobe width contracts systematically, confirming a predictable and monotonic relationship: a larger capacitor requires more charge to change its voltage, $v_{C}$, thereby reducing the rate of memristance modulation within a signal period and leading to a less pronounced hysteresis loop. Conversely, a smaller capacitor enables faster state transitions, yielding a wider lobe and stronger nonlinearity. This inverse relationship provides a practical and precise design parameter for tailoring the emulator’s dynamic resistance range. For instance, in a biomedical front-end system requiring adaptive gain control, C can be selected or made programmable to adjust the signal conditioning bandwidth according to the frequency content of incoming biosignals, enabling real-time optimization of noise filtering and dynamic range.

The operational stability of the DMEC across environmental temperature variations is critical for practical applications, particularly in wearable or implantable biomedical systems. The circuit was simulated across a temperature range from −25 °C to 75 °C, as shown in Figure 5b. The results demonstrate exceptional thermal stability: the pinched hysteresis loops for all temperatures are nearly superimposed, with only minimal variation in the current magnitude and no observable distortion in the lobe shape or symmetry. This indicates that the competing temperature-dependent effects in the DTMOS transistors, the reduction in carrier mobility (μ) and the decrease in threshold voltage, effectively counterbalance each other in this specific topology. Consequently, the net channel current and, more importantly, the state-modulation dynamics governed by capacitor C remain stable. This inherent thermal robustness ensures that the DMEC’s memristive function is reliably maintained over a wide temperature span without requiring external compensation, a significant advantage for low-power, integrated biomedical interfaces. The linearity and dynamic range of the DMEC were characterized by sweeping the input sinusoidal amplitude A from 1.2 V to 2.0 V at a fixed frequency of 10 MHz. The resulting pinched hysteresis loops, shown in Figure 5c, demonstrate the circuit’s consistent memristive operation across this voltage range. While the hysteresis lobe area exhibits dependence on input amplitude, the relationship is more complex than a simple linear correlation. At lower amplitudes (A=1.2 V), the loop remains well-defined but with reduced lobe width, indicating moderate state modulation. As the amplitude increases, the injected charge per cycle grows, enhancing the swing in the capacitor voltage, $v_{C}$, and thereby strengthening the resistance modulation effect. This manifests as an expansion of the hysteresis lobe, confirming the expected amplitude-dependent behavior. Crucially, the pinching at the origin is maintained across all amplitudes, preserving the fundamental memristive fingerprint. For biomedical front-end applications, this amplitude sensitivity can be strategically employed in adaptive circuits, to implement compressive sensing where larger biosignal amplitudes naturally experience higher attenuation through increased memristance, effectively auto-scaling the system’s dynamic range. Additionally, to validate the DMEC’s functionality as a true floating, two-terminal circuit element, its behavior was tested in fundamental network configurations: standalone, series, and parallel connections, as illustrated in Figure 5d. When two identical DMECs are connected in series, the composite PHL exhibits a current scaling consistent with a doubled memristance, as the same current flows through both devices with additive voltage drops. In a parallel configuration, the combined PHL shows a current doubling for a given voltage, effectively halving the equivalent memristance. These results confirm that the proposed emulator obeys the same series and parallel combination rules as a theoretical passive memristor. This property is essential for constructing more complex memristive networks and arrays for advanced analog processing within an integrated biomedical sensor interface.

**Figure 6:**
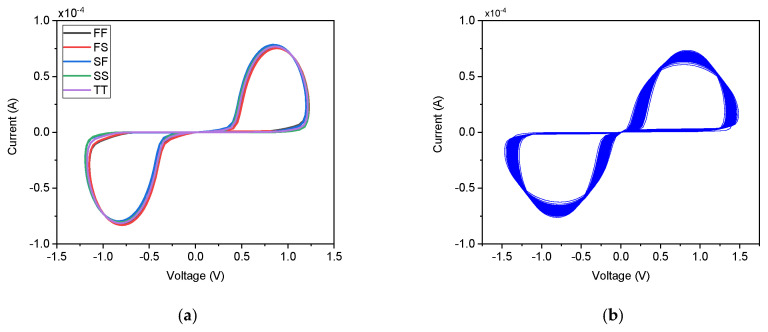
(**a**) Process corner and, (**b**) Monte Carlo analysis of the DMEC.

**Figure 7:**
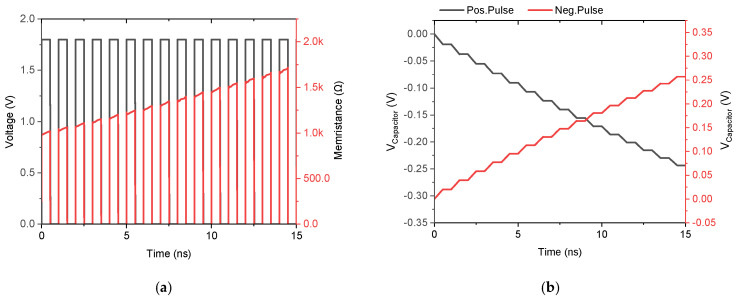
Non-volatile behavior of the proposed DMEC with 12 pF capacitor under 10 ns pulse excitation. (**a**) Memristance variation with +1.8 V pulses and (**b**) capacitor voltage, $v_{C}$, with ±1.8 V bipolar pulses showing bidirectional state programming and retention between pulses.

The robustness and manufacturability of the DMEC were evaluated through comprehensive process corner analysis and Monte Carlo simulations incorporating both global process variations and local device mismatch. As depicted in Figure 6a, the circuit was simulated across five critical process corners: fast–fast (FF), fast–slow (FS), slow–fast (SF), slow–slow (SS), and typical–typical (TT). All corners exhibit well-defined pinched hysteresis loops. All five corners maintain the fundamental memristive fingerprint, the hysteresis loop remains pinched at the origin with preserved lobe symmetry. All corners exhibit well-defined pinched hysteresis loops with remarkably low variation in both current magnitude and lobe width. This stability stems from the self-compensating nature of the DTMOS-based design, while process variations affect the absolute threshold voltage and mobility, the body-effect coupling in the DTMOS transistors ensures that the state-dependent resistance modulation, governed by capacitor voltage, $v_{C}$, remains relatively invariant. The TT corner serves as the reference, while the FF corner shows a marginal increase in current due to enhanced carrier mobility, and the SS corner shows a slight reduction. To further quantify variability, 200-run Monte Carlo simulations were performed. As depicted in Figure 6b, all 200 iterations successfully produce the characteristic hysteresis. This confirms the DMEC’s excellent robustness against both systematic process variations and random device mismatches, ensuring reliable performance for integrated implementation in biomedical CMOS processes. The non-volatile nature, the ability to retain its resistance state after the input signal is removed, was tested by applying a pulsed voltage train. Figure 7a plots the applied positive pulse alongside the corresponding memristance variation. Upon application of the positive pulse, the memristance exhibits a near-instantaneous increase, tracking the input stimulus. When the input pulse returns to zero, the memristance does not reset immediately but instead decays gradually, maintaining a significant portion of its charged state. The output current and thus the memductance stabilizes at a value different from its initial state and maintains this value until the next pulse arrives. The non-volatile nature of the memristor emulator depends on the capacitor. Figure 7b demonstrates the response to alternating polarity pulses (±1.8 V). As shown, the capacitor voltage increases during positive pulses and decreases during negative pulses, confirming bidirectional state programming. Importantly, in the absence of an input pulse, during the interval between pulses, the capacitor voltage remains constant, indicating that the memristor emulator retains the previous state information. This charge retention on the capacitor emulates the non-volatile behavior of an actual physical memristor, where the device resistance persists without power supply. Additionally, the retention time is proportional to the capacitor value, with larger capacitors providing longer retention due to slower discharge through leakage currents. Therefore, the proposed DMEC exhibits short-term capacitive retention rather than true non-volatility. As a CMOS-based emulator, the state is stored as charge on a physical capacitor, which will eventually discharge through leakage paths such as junction leakages, subthreshold conduction, and parasitic resistances. This is an inherent limitation of all CMOS-based memristor emulators that use capacitors as the state-holding element.

The simulation results collectively confirm that the proposed two-DTMOS, one-capacitor emulator successfully replicates all fundamental fingerprints of a floating memristor. The circuit exhibits a clearly pinched, frequency-dependent hysteresis loop, electronically tunable memristance, non-volatile state retention, and robust operation across temperature, amplitude, and process variations. Statistical Monte Carlo analysis further validates its manufacturability, with minimal performance deviation across 200 runs. These characteristics demonstrate the DMEC’s suitability as a compact, low-power, and reliable building block for integrated neuromorphic and adaptive analog systems, particularly within a resource-constrained biomedical front-end.

Furthermore, the simulations presented in this work were performed at the schematic level using the foundry-supplied MOSFET models for the 0.18 μm CMOS process. These models inherently include intrinsic transistor parasitics such as gate capacitances, junction capacitances, overlap capacitances and first-order parasitic effects captured within the MOSFET framework. However, they do not include several parasitic elements that only become available after physical layout and extraction, namely, extracted parasitic capacitances from interconnect routing, parasitic resistances of metal lines and vias, fringe capacitances between adjacent metal traces, substrate coupling effects, and MIM capacitor bottom plate parasitics. These additional parasitics can significantly influence circuit behavior at frequencies approaching 500 MHz and therefore represent an important consideration for future validation. Post-layout simulations incorporating these extracted parasitics are planned as part of our ongoing work to provide a more accurate assessment of the high-frequency performance limits.

**Figure 8:**
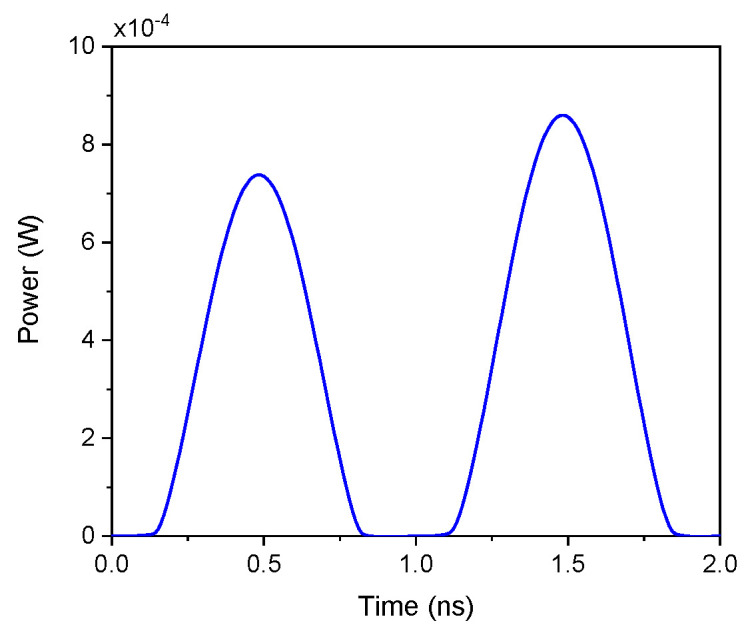
Power consumption of the DMEC.

The dynamic power consumption of the proposed DMEC was evaluated for the maximum operating frequency to assess its suitability for low power. Figure 8 illustrates the simulated dynamic power as a function of time. The dynamic power was calculated using the time-domain integral of the instantaneous power over one complete cycle using the following expression:

$$P_{dynamic}=\frac{1}{T}\int_{0}^{T}v_{AB}(t)\cdot i_{AB}(t)\;dt$$

where T is the period of the input signal. At the maximum simulated frequency of 500 MHz, the dynamic power consumption is 318 μW.

Additionally, for the proposed DMEC, the energy per cycle was calculated at two representative frequencies spanning the operating range. At 100 kHz, corresponding to a period of 10 μs, the energy dissipated per cycle is 107.7 pJ. At the maximum simulated frequency of 500 MHz, with a period of 2 ns, the energy per cycle reduces significantly to 6.36 pJ. This frequency-dependent decrease in energy dissipation is consistent with the characteristic memristive behavior observed in Figure 4, where the hysteresis lobe area progressively contracts as frequency increases. The substantial reduction in energy per cycle, from 107.7 pJ at low frequency to 6.36 pJ at high frequency, demonstrates that the DMEC maintains energy efficiency across its entire operating range while preserving memristive functionality.

### 3.2. Experimental Results

**Figure 9:**
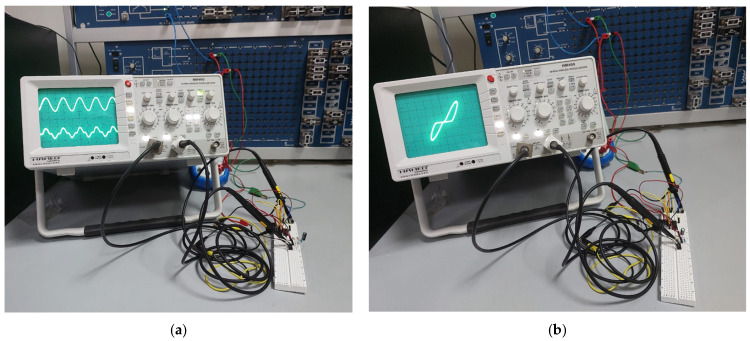
Experimental validation. (**a**) Measured transient response and (**b**) pinched hysteresis loop at 500 Hz.

To validate the simulated performance and demonstrate the practical feasibility of the proposed DTMOS-based memristor emulator, an experimental prototype was constructed and tested. The core of the prototype utilizes a CD4007UB CMOS-integrated circuit, which contains complementary pairs of MOSFETs suitable for emulating the DTMOS configuration by externally connecting the gate and substrate terminals. A small, precise series resistor was incorporated into the circuit to enable current measurement by sensing the voltage drop across it. The complete experimental setup, including the CD4007UB, the external state capacitor C, the signal generation unit, and the measurement instrumentation, is illustrated in Figure 9. The transient voltage response across the DMEC and the series resistor was captured directly using a digital oscilloscope. Figure 9a displays this measured transient response for a sinusoidal input. Subsequently, the acquired voltage and current data were processed to plot the current–voltage characteristic. Figure 9b presents the corresponding pinched hysteresis loop obtained for an input frequency of 500 Hz and a state capacitor C=1 μF. This discrete implementation successfully verifies the memristive behavior under real-world conditions, bridging the gap between simulation and potential integrated circuit realization.

**Figure 10:**
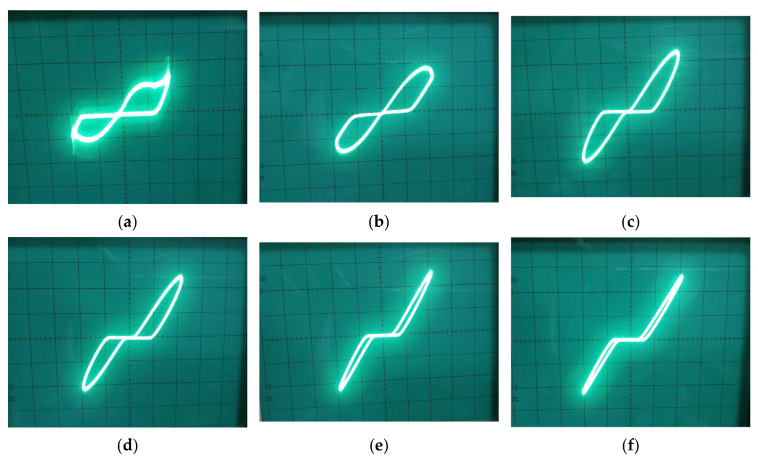
PHLs at (**a**) 100 Hz, (**b**) 250 Hz, (**c**) 500 Hz, (**d**) 800 Hz, (**e**) 1.2 KHz and, (**f**) 1.5 KHz.

**Figure 11:**
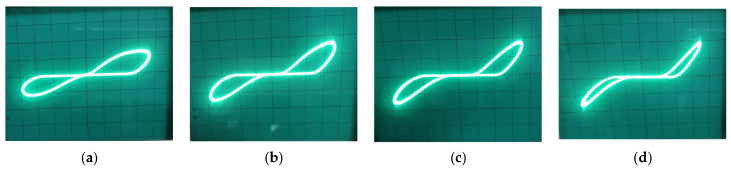
PHLs at (**a**) 100 kHz, (**b**) 200 KHz, (**c**) 500 KHz, (**d**) 800 KHz.

The frequency-dependent contraction of the hysteresis lobe was experimentally validated across two distinct operating regimes by adjusting the state capacitor C. In the first setup, employing a large capacitor (C=1 μF) to establish a long time constant, the input frequency was swept from 100 Hz to 1.5 kHz. As shown in Figure 10, the PHL at 100 Hz exhibits a wide, well-defined lobe, indicating strong state modulation. As frequency increases, the lobe area contracts progressively; at 1.5 \kHz, the loop is notably narrower, demonstrating the inverse relationship between hysteresis area and frequency when the circuit’s intrinsic time constant is dominated by the large capacitor. In the second setup, the capacitor was reduced to C=500 nF. The frequency was subsequently swept from 100 kHz to 800 kHz. The results, plotted in Figure 11, confirm the same contraction trend in a much higher band. At 100 kHz, a clear hysteresis lobe is present. As frequency approaches 800 kHz, the lobe area diminishes significantly, and the I–V characteristic begins to linearize. The consistent behavior with different C values confirms the governing role of the circuit’s time constant, providing a straightforward design rule for tailoring the emulator’s dynamic response for specific applications, from low-frequency biosignal conditioning to higher-frequency analog processing.

The experimental validation using the discrete CD4007UB prototype successfully demonstrates the memristive behavior from 100 Hz to 800 kHz, confirming the functional correctness of the proposed topology under real-world conditions. The frequency-dependent contraction of the hysteresis lobe follows the same trend observed in simulation, validating the underlying operating principle described. It is important to note that the 800 kHz upper limit of experimental validation is imposed by the inherent frequency limitations of the discrete CD4007UB component, not by the proposed circuit topology itself. The CD4007UB is fabricated in a legacy bulk CMOS process with characteristics that inherently limit high-frequency operation, including a typical transition frequency of only 10–50 MHz for minimum-size devices, large parasitic pad and package capacitances (several pF) that dominate at higher frequencies, limited ability to implement a true DTMOS configuration due to the shared substrate connection in the discrete package, and significant parasitic inductances from bond wires and PCB traces that introduce impedance mismatches as frequency increases. These factors collectively restrict any circuit implemented with this discrete component to the low MHz range, regardless of the topology employed. Consequently, the 800 kHz experimental bandwidth represents the limit of the discrete prototype, not the potential of the proposed design.

The simulated 500 MHz maximum frequency represents the expected performance of the proposed DMEC when implemented in a standard 0.18 μm CMOS process. This projection is supported by the theoretical analysis showing inverse frequency dependence of the hysteresis lobe area, the high transition frequency of minimum-length devices in 0.18 μm CMOS, typically exceeding 50 GHz, and literature precedent where similar minimalist topologies have demonstrated operation in the hundreds of MHz range when implemented in advanced CMOS nodes [9,22,23]. Furthermore, the experimental validation up to 800 kHz with the discrete prototype confirms that the circuit operates as theoretically predicted across a frequency range, providing confidence that the same operating principles will extend to higher frequencies in an integrated implementation. Future work will focus on fabricating the proposed DMEC in a 0.18 μm CMOS process to experimentally validate the high-frequency performance predicted by simulation.

## 4. Comparison and Discussion

This section provides a comparative analysis of the proposed two-DTMOS memristor emulator with notable recent works in the literature, as summarized in Table 1. The comparison focuses on key metrics for practical integration: circuit complexity, operating performance, and power efficiency. Circuit complexity, directly impacting silicon area and parasitic effects, is affected by the number of active and passive components. As Table 1 illustrates, the proposed DMEC employs only two MOSFETs, configured as DTMOS, and one capacitor, representing one of the most minimalist topologies reported. This contrasts with several designs requiring three [16,21,23,24,25] or four [14,15,19,20,22,26,27] transistors, often with additional resistors [18,28] or more complex capacitor networks. The reduction to two active devices is achieved by exploiting the DTMOS transistors to perform dual functions: they act as both the state-dependent variable resistor and the control switches for the capacitor. This intrinsic multifunctionality eliminates the need for separate biasing or control stages, significantly simplifying the layout and enhancing its suitability for dense, low-power integrated systems, such as biomedical sensor arrays.

Performance is evaluated based on operating frequency, functional configurability (floating/grounded), and experimental validation. The proposed DMEC demonstrates a competitive maximum operating frequency of 500 MHz in simulation, aligning with high-performance designs like [24] (50 GHz, though grounded) and exceeding many in the 10–150 MHz range [12,22,25,27,33]. This high-frequency capability stems from the minimalist topology, which reduces internal node parasitics. Furthermore, the DMEC is verified as a floating emulator, a more versatile and challenging configuration than a grounded one, as it can be inserted anywhere in a circuit without a fixed bias reference. Additionally, the work is validated through both simulation and experimental measurement, confirming its practical viability, a step not always taken in works reporting very high frequencies [28,29].

Power consumption is a critical metric for wearable and implantable applications. The DMEC is designed for zero static power consumption, as it requires no external DC bias voltages or currents; it is driven solely by the input signal. This passive operating principle places it among the most power-efficient designs in the comparison, such as those of [20,23,24,25,28,32], which also report zero or negligible static power. It notably outperforms emulators that consume power in the µW to mW range [9,22,27,30,31,33]. The achieved combination of zero static power, two-transistor complexity, and high-frequency floating operation is a distinctive advantage of the proposed architecture.

The comparative analysis establishes that the proposed DMEC occupies a competitive position in the multi-dimensional design space defined by component count, operating frequency, and power efficiency. Unlike prior works that often optimize for a single metric, such as ultra-high frequency [24] or exceptionally low power [33], this work delivers a balanced and a combination of key advantages. Specifically, the proposed architecture achieves a simultaneous minimization of active components and static power consumption (zero bias), while maintaining a high operating frequency of 500 MHz and providing the flexibility of floating operation. These performance characteristics are not only simulated but are validated by experimental measurements from a discrete prototype. Consequently, this minimalism, power efficiency, high maximum operation frequency, and versatility render the DMEC an exceptionally suitable candidate for integration into the analog front ends of next-generation, resource-constrained systems, such as wearable and implantable biomedical monitors, where silicon area, energy budget, and adaptive signal conditioning are critical constraints.

The promising results of this work motivate focused future research to transition the DMEC from a discrete emulator to an integrated, system-ready component for biomedical electronics. The primary objective will be the design and fabrication of the emulator in a dedicated CMOS microchip. A full-custom integrated circuit will achieve true area efficiency, as the two DTMOS transistors and a MOS capacitor can be realized in an extremely compact layout, likely occupying very low area. This minimal footprint ensures that the emulator adds negligible area overhead to a complete biosensor interface chip. System-level research will then target integration into ultra-low-power analog front-end architectures for specific biomedical modalities. The DMEC can be integrated at multiple points within a typical biomedical analog front-end to provide adaptive functionality. At the input stage, the DMEC can be placed in series with the electrode to provide dynamic impedance matching for long-term monitoring applications such as EEG and ECG, where electrode–skin contact impedance can vary due to sweating, movement, or drying of conductive gel. The DMEC’s memristance automatically adjusts based on the signal history, compensating for these impedance variations and mitigating motion artifacts without requiring additional control circuitry, improving signal quality while maintaining the zero-static-power advantage of the emulator. In the amplification stage, the DMEC can replace a fixed resistor in the feedback network of an instrumentation amplifier to provide automatic gain control. As the input signal amplitude varies from μV-range EEG signals to mV-range ECG signals, the DMEC’s memristance automatically adjusts, eliminating the need for power-hungry control loops. In the filtering stage, the DMEC can be integrated into active filter topologies to create adaptive frequency response. By replacing a resistor with the DMEC, the cutoff frequency becomes $f_{c}=W/(2\pi C)$, where W is the memductance, allowing the filter bandwidth to adapt to the signal’s frequency content, narrowing when noise is present and widening when faithful signal reproduction is needed. For EEG applications, this enables adaptive notch filters that can track and reject time-varying power-line interference (50/60 Hz), while for ECG processing, it allows the filter bandwidth to adjust based on heart rate variations.

Beyond direct signal conditioning, the DMEC can also be employed to implement a tunable ring oscillator for on-chip clock generation in biomedical systems. By replacing a fixed resistor in the oscillator circuit with the DMEC, the oscillation frequency becomes dependent on the memristance value, enabling several critical functions. First, programmable sampling rates allow the ADC sampling frequency to be dynamically adjusted based on input signal characteristics, higher sampling rates for detailed analysis of transient events, and lower rates for power saving during idle periods. Second, adaptive clock generation enables the system clock to self-tune to optimal frequencies for different operational modes, reducing power consumption when full performance is not required. Third, in wireless biomedical implants, the DMEC-based oscillator can modulate the carrier frequency for data transmission, with the memristance controlled by the biosignal itself. Fourth, the zero-static power characteristic makes the DMEC ideal for always-on wake-up receivers that monitor for incoming signals while consuming minimal power. This tunable oscillator implementation is particularly valuable in ultra-low-power biomedical devices where clock generation must be both energy efficient and adaptable to varying operational requirements. Furthermore, exploring the circuit’s behavior in networked configurations presents a compelling direction for neuromorphic engineering. Connecting multiple DMECs to form small-scale synaptic arrays could enable the investigation of more complex functions such as pattern learning, temporal signal processing, and computing. This would evolve the component from a standalone memristor emulator into a foundational building block for brain-inspired computing systems, opening new possibilities for hardware implementation of neural networks and adaptive signal processing architectures.

## 5. Conclusions

This research has demonstrated the design, analysis, and validation of an ultra-compact floating memristor emulator. The proposed DTMOS-based circuit, employing only two transistors and one capacitor, fundamentally simplifies the hardware implementation of memristive behavior. Mathematical modeling derived the state dynamics and frequency-dependent memductance, confirming the circuit’s conformity to memristive system theory. Simulation results verified all key fingerprints: a pinched hysteresis loop scalable with frequency and capacitance, short-term non-volatility, and stable operation under environmental and manufacturing variations. Practical functionality was proven through experimental measurement, bridging simulation and real-world performance. The proposed emulator stands out by concurrently minimizing component count, eliminating static power, and maintaining high-frequency floating operation, a combination rarely achieved in prior art. Consequently, this work provides a foundational, efficient, and integrable memristor emulator core, paving the way for its adoption in adaptive analog circuits for next-generation low-power biomedical interfaces and neuromorphic processing systems.

## Figures and Tables

**Table 1:** Comparison with existing CMOS memristor emulator circuits.

Ref.	MOSFET Count	Passive Components	Floating/ Grounded	Frequency	Technology	Power Consumption	Experiment/Simulation
[29]	4	0	Grounded	100 MHz	0.18 µm	Nil	Simulation
[30]	4	1-C	Both	100 MHz	65 nm	75 μW	Both
[22]	2	1-C	Both	150 MHz	65 nm	13.1 μW	Both
[24]	2	1-C, 1-R	Grounded	50 GHz	45 nm	0	Both
[26]	4	1-C	Floating	3 MHz	0.18 µm	8.24 µW	Both
[23]	4	1-C	both	500 MHz	0.18 µm	0	Both
[27]	3	1-C	Both	50 MHz	0.18 µm	57.4 μW	Both
[28]	4	0	both	250 MHz	0.18 µm	0	Simulation
[25]	3	0	both	30 MHz	0.18 µm	0	Both
[31]	4	0	Grounded	100 KHz	0.18 µm	40 µW	Both
[32]	3	1-C	Grounded	100 KHz	0.18 µm	0	Both
[12]	4	0	Floating	50 MHz	90 nm	2.6 µW	Both
[33]	1	1-C, 1-R	both	80 MHz	45 nm	7.75 pW	Both
[20]	3	1-C	Grounded	24 MHz	90 nm	0	Both
[9]	2	0	Grounded	300 MHz	65 nm	963 μW	Both
This work	2	1-C	Both	500 MHz	0.18 µm	0	Both

## Data Availability

Data are contained within the article.
